# Misframed ubiquitin and impaired protein quality control: an early event in Alzheimer’s disease

**DOI:** 10.3389/fnmol.2015.00047

**Published:** 2015-09-02

**Authors:** Romina J. Gentier, Fred W. van Leeuwen

**Affiliations:** Department of Neuroscience, Faculty of Health, Medicine and Life Sciences, Maastricht UniversityMaastricht, Netherlands

**Keywords:** frameshift mutation, mRNA surveillance, molecular misreading, proteasome, amyloid precursor protein, tau, neurodegeneration

## Abstract

Amyloid β (Aβ) plaque formation is a prominent cellular hallmark of Alzheimer’s disease (AD). To date, immunization trials in AD patients have not been effective in terms of curing or ameliorating dementia. In addition, γ-secretase inhibitor strategies await clinical improvements in AD. These approaches were based upon the idea that autosomal dominant mutations in amyloid precursor protein (APP) and Presenilin 1 (PS1) genes are predictive for treatment of all AD patients. However most AD patients are of the sporadic form which partly explains the failures to treat this multifactorial disease. The major risk factor for developing sporadic AD (SAD) is aging whereas the Apolipoprotein E polymorphism (ε4 variant) is the most prominent genetic risk factor. Other medium-risk factors such as triggering receptor expressed on myeloid cells 2 (TREM2) and nine low risk factors from Genome Wide Association Studies (GWAS) were associated with AD. Recently, pooled GWAS studies identified protein ubiquitination as one of the key modulators of AD. In addition, a brain site specific strategy was used to compare the proteomes of AD patients by an Ingenuity Pathway Analysis. This strategy revealed numerous proteins that strongly interact with ubiquitin (UBB) signaling, and pointing to a dysfunctional ubiquitin proteasome system (UPS) as a causal factor in AD. We reported that DNA-RNA sequence differences in several genes including ubiquitin do occur in AD, the resulting misframed protein of which accumulates in the neurofibrillary tangles (NFTs). This suggests again a functional link between neurodegeneration of the AD type and loss of protein quality control by the UPS. Progress in this field is discussed and modulating the activity of the UPS opens an attractive avenue of research towards slowing down the development of AD and ameliorating its effects by discovering prime targets for AD therapeutics.

Alzheimer’s disease (AD) is a progressive multifactorial neurological disease and the most prevalent form of dementia, accounting for 60–80% of all cases of dementia (Barnes and Yaffe, 2011). Many reviews have been written about this complex neurodegenerative disease e.g., (Selkoe et al., 2012). The sporadic (SAD) and familial (FAD) forms are the two major types of AD which differ in the age and cause of onset (Figure 1). SAD is heterogeneous with risk factors for developing AD including aging, cognitive inactivity, depression, oxidative stress, brain injury, epigenetic factors and mild cognitive impairment (MCI). Cardiovascular factors (cholesterol status, diabetes, midlife hypertension, obesity, physical inactivy and smoking) as well as genetic risk or dubbed timing factors (apolipoprotein E4 polymorphism; Apoε4) are also associated with the development of SAD (Querfurth and LaFerla, 2010; Alzheimer’s Association, 2012). Other medium-risk factors such as a rare missense mutation (R47H subsitiution) in the gene encoding the triggering receptor expressed on myeloid cells 2 (TREM2), which has in a normal situation an anti-inflammatory role in the brain, lead to an increased risk for AD through aberrant inflammatory processes (Jonsson et al., 2013). An additional nine low risk factors detected via Genome Wide Association Studies (GWAS) were reported to contribute as well to AD (Holton et al., 2013). Genetic factors associated with AD consists of mutations in the genes encoding for the amyloid precursor protein (APP; chromosome 21), Presenilin 1 (PS1; chromosome 14) and Presenilin 2 (PS2; chromosome 1).

**Figure 1:**
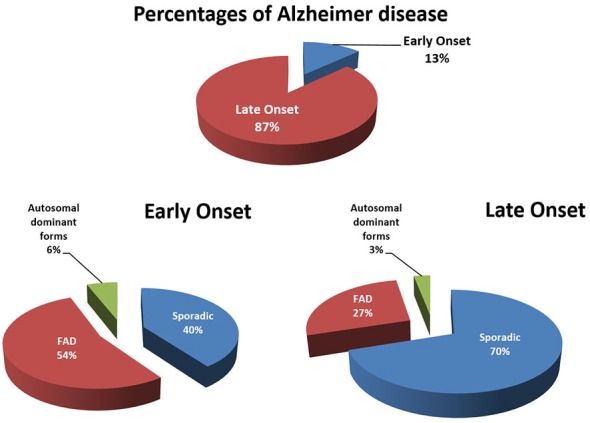
**Early [<65 years; early-onset AD (EOAD)] and late onset [>65 years; late-onset AD (LOAD)] forms of Alzheimers disease (AD) subdivided in familial, sporadic and autosomal dominant forms of AD.** “Familial” means that AD was observed in relatives of the first degree. In familial EOAD, the majority (54%) is not yet linked to a chromosome, whereas 6% is inherited in an autosomal dominant way and linked to chromosome 14 (PS1), chromosome 1 (PS2) and chromosome 21 amyloid precursor protein (APP; Harvey et al., 2003; Mercy et al., 2008). In familial LOAD, the majority is not yet linked to a chromosome, whereas a minority is inherited in an autosomal dominant way. A subset has been linked to chromosome 12 (Pericak-Vance et al., 1997). Figure was updated in 2015.

A large number of pathogenic mechanisms in AD have been identified (Jellinger, 2009) including proteasomal dysfunction, oxidative stress, mitochondrial dysfunction, fragmentation of Golgi complex, cellular/axonal transport disruption, mutation of molecular chaperones, dysfunctional neurotrophins, neuroinflammatory processes and more recently seeding (Jucker and Christen, 2013). All these complex mechanisms are interconnected resulting in cell dysfunction, neuron shrinkage and cell death such as in parts of the hippocampal complex and locus coeruleus (Mann et al., 1980; West et al., 1994; Heneka et al., 2006). It needs to be noted that neuron loss is not a widespread phenomenon in AD (Regeur et al., 1994) and that cell shrinkage, resulting in weight loss of the brain, erroneously suggested neuron loss, is occurring (Hoogendijk et al., 1995; Lee et al., 2015). The present review focusses on two mechanisms mentioned above; proteasomal dysfunction by a mutant form of ubiquitin B (UBB^+1^) and partially in combination with oxidative stress (Hope et al., 2003; Irmler et al., 2012; Braun et al., 2015), both contributing to disease-specific inclusions (van Leeuwen et al., 1998; Dennissen et al., 2012).

The neuropathogenesis of AD includes so called negative and positive lesions. Negative lesions describe the loss of cholinergic neurons in the brain, whereas positive lesions are the accumulation of abnormal/misfolded proteins known as deposits (Serrano-Pozo et al., 2011). This accumulation of putative toxic protein species in the brain of the patients is one common hallmark of many neurodegenerative diseases, such as AD and, as well, Parkinson’s (PD) and Huntington’s (HD) diseases (Fischer et al., 2003). These three diseases belong to a group sharing the common feature of the accumulation of insoluble protein deposits in neurons and are now designated “conformational diseases” coined as such (Lomas and Carrell, 2002). AD is associated with progressive accumulation of two hallmarks, namely extracellular Amyloid β (Aβ) plaques and intracellular neurofibrillary tangles (NFTs; Selkoe, 2001; Duyckaerts et al., 2009) Currently there are no indications of increased Aβ and tau protein production in AD which suggests that the accumulation of these proteins is caused by a lack of cellular clearance (Olsson et al., 2003). The degradation pathway of these proteins remains elusive but autophagy is an attractive possibility (Lee et al., 2013; Nilsson and Saido, 2014). Although Aβ and tau do not seem to be substrates of the 26S proteasome, both proteins are able to impair functioning of the 26S proteasome (Saido and Leissring, 2012). Further details concerning Aβ, tau, and the proteasome are given in the following paragraphs.

## The Aβ Peptide Hypothesis

**Figure 2:**
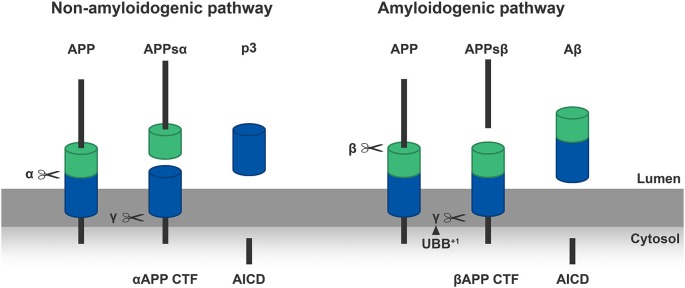
**Left panel: α-secretase (α) cleaves the APP molecules inside the Aβ sequence in a non-amyloidogenic manner, creating a soluble N-terminal part of APP (APPsα) and a C-terminal part (αAPP CTF) which is anchored in the membrane.** Subsequently the γ-secretase (γ) cleaves the C-terminal part in a p3 peptide and an APP intracellular domain (AICD). **Right panel**: β-secretase (β) cleaves the APP at the N-terminus of the Aβ-sequence in an amyloidogenic manner, generating an N-terminal fragment of APP (APPsβ) and a C-terminal part (βAPP CTF). Following, the γ-secretase (γ) cleaves the βAPP-CTF which results in Aβ and AICD. We showed that ubiquitin B^+1^ (UBB^+1^) is able to modulate the formation of Aβ plaques in the transgenic (tg) line via γ-secretase, this is shown in the **Right panel** by the arrowhead (van Tijn et al., 2012; Gentier et al., 2015b).

The amyloid hypothesis focuses on the endoproteolytic cleavage pathway of APP, a transmembrane protein (Glenner and Wong, 1984). This “hypothesis” is supported by the fact that Aβ plaques are present in AD brains and that mutations in the APP gene are involved in the autosomal dominant inherited form of AD (Masters and Selkoe, 2012). APP is a precursor molecule which experience proteolysis to generate Aβ species of different length. Normally, a cascade of endoproteolytic cleavages of APP by α-, β- and γ-secretases results in the production of both non-amyloidogenic (the α-secretase pathway) and amyloidogenic (β-secretase pathway) peptides (Figure 2). The γ-secretase cleavage is a heterogeneous event, so depending on the site of cleavage by this protease Aβ-peptides of different size are generated: Aβ_40_ and Aβ_42_ are the most common ones (Haass et al., 2012). The two additional amino acids in Aβ_42_ compared to Aβ_40_ render Aβ_42_ more hydrophobic thereby making it more susceptible aggregate. Due to a higher rate of insolubility and fibrillization, Aβ_42_ is more abundant than Aβ_40_ in extracellular plaques. In many types of AD the ratio of Aβ_42_/Aβ_40_ is increased (Masters and Selkoe, 2012). This ratio can also be influenced by mutations in the α-secretase (ADAM 10), favoring the amyloidogenic pathway (Suh et al., 2013). Aβ peptides eventually oligomerize to fibrils which eventually accumulate intracellularly, are secreted and promote synaptotoxicity by seeding (Duyckaerts et al., 2009). Three major types of Aβ plaques can be distinguished in AD: diffuse amyloid plaques, dense-core plaques and neuritic plaques (NPs; Serrano-Pozo et al., 2011). Currently, there is still an ongoing debate about which species is exactly more toxic: intracellular oligomers or extracellular plaques? A lot of mutations are found within the γ-secretase complex (PS1, PS2) which cause excessive production of Aβ42. Excessive accumulation of Aβ species and subsequent seeding is the initiating event in the pathogenesis of AD according to this hypothesis. It turned out that the formation of Aβ plaques in a transgenic (tg) model of AD can be modulated by UBB^+1^ expression via γ-secretase and of this multimeric complex at least the presenilin expression (van Tijn et al., 2012; Gentier et al., 2015b).

Other studies showed genomic duplications in the APP locus in families suffering from early-onset AD (EOAD) with concurrent cerebral amyloid angiopathy. These data suggests as well that increased expression of APP has an important role in the AD disease etiology (Rovelet-Lecrux et al., 2006; Sleegers et al., 2006). In addition, the link between Down syndrome (DS) and AD regarding neuropathogenesis and dementia, supports the relevance of Aβ even further, because DS patients have three copies of chromosome 21 and APP is located at the same chromosome. In DS, Aβ plaques already appear around 30 years of life e.g., (van Leeuwen et al., 2006). Development of dementia in DS patients is the result of overproduction of toxic Aβ species as a direct consequence of the triplication of the APP gene (Weksler et al., 2013). In addition, a coding mutation (A673T) in the APP gene was found in an Icelandic population which protects against AD and cognitive decline in the elderly without AD. This A673T mutation results in an approximately 40% reduction in the formation of amyloidegenic peptides *in vitro* and provides proof of principle for the hypothesis that reducing the amyloidogenic cleavage of APP may protect against AD (Jonsson et al., 2012). At the present time, evidence is appearing which suggests that Aβ is neither sufficient nor necessary in the development of AD but only related to it. This finding is based on the observation that the amount and distribution of Aβ accumulation do not correlate with the degree of cognitive impairment and that Aβ is present as well in the brains of cognitively normal elderly people (Drachman, 2014). The prevalence of amyloid pathology increased from age 50 to 90 years from 10% (95% CI, 8–13%) to 44% (95% CI, 37–51%) among participants with normal cognition (Jansen et al., 2015). In addition, Aβ immunization trials in AD patients have been up to now unsuccessful in terms of curing or ameliorating dementia probably because these clinical trials started too late in the disease process and even resulted in meningoencephalitis in 6% of the cases. e.g., (Holmes et al., 2008; Lannfelt et al., 2014). A limited clearance of pre-existing Aβ plaques was also shown in experimental studies with the same tg AD mouse model as used for our studies (Tucker et al., 2008; van Tijn et al., 2012). As mentioned before using line 85 we were able to lower the formation of Aβ plaques. This shows again that an earlier start of immunization trials is an option (Reiman et al., 2012). Another strategy to lower the amount of Aβ plaques or signs of dementia is the use of γ-secretase inhibitors that were unsuccessful so far, because these trials were halted due to worsening of the outcomes (De Strooper, 2014). A detailed discussion on these trials and the biology of APP processing goes beyond the scope of the present review.

## The Tau Hypothesis

This so-called “Tau and tangle hypothesis” suggests that hyperphosphorylated tau is the primary causative factor in AD development leading to neurotoxicity. This hypothesis is based on the observation that NFT density and distribution correlates with the clinical stage of the disease (Morris et al., 2011; Braak and Del Tredici, 2013).

The tau gene (located on chromosome 17) is translated into a protein found predominantly in nerve cells, concentrated in axons, in normal brain and the protein has six isoforms. It contains a microtubule-binding domain formed out of three or four repeating regions (tau 3 R and tau 4 R) (Goedert et al., 2012). The history of Tau has been reviewed recently but mutations in the Tau gene have only be shown for tauopathies related to AD (Mandelkow and Mandelkow, 2012). Tau initiates and stabilizes neuronal microtubules, which are components of the cytoskeleton, by binding tubulin in healthy brain (Iqbal et al., 2009; Kadavath et al., 2015). When tau is hyperphoshorylated, the ability to bind microtubules is reduced. Accumulations of hyperphosphorylated and misfolded tau proteins are observed in affected neurons in AD. Under normal conditions tau is a soluble protein but it becomes insoluble in its hyperphosphorylated state. In contrast to the normal function of tau protein, the aberrant protein causes disruption of microtubules, dysregulated axonal transport, sequestration of normal tau and subsequently promotes self-assembly (Simic et al., 2009). Three kinds of tau aggregates can be differentiated in AD: pretangles and NFTs in the cell body of neurons, neuropil threads (NTs) in the dendrites, and dystrophic neurites associated with NPs in the axons (Duyckaerts et al., 2009). These different forms of tau are detected in our studies by using the MC1 antibody, recognizing aberrant tau in pretangles, and CP13, recognizing phosphorylated tau and appeared to coincide with the appearance of UBB^+1^. How UBB^+1^ is related to aberrant and phosphorylated Tau, both spatially and temporally needs to be determined. A common procedure to classify the degree of AD pathology is the so-called Braak staging (Stages I–VI) based on topographical and time-dependent distribution of NFTs and NTs (Braak and Braak, 1991). It was generally accepted that the progression of tau pathology follows a consistent pattern of propagation, which initiates in the transentorhinal cortex and eventually affects all the subdivisions of the neocortex (Braak and Braak, 1995). Currently, however, more evidence is available suggesting that tau aggregation starts in other regions of the brain, such as the brainstem, in particular the locus coeruleus (Braak and Del Tredici, 2011). Based up on these data now also tau immunizations have started (e.g., Sankaranarayanan et al., 2015).

## The Ubiquitin Proteasome System and UBB^+1^

The levels of many proteins must be highly regulated both spatially and temporally in order for cellular functions to proceed accurately (Hegde et al., 2014). The ubiquitin proteasome system (UPS) is one of the major intracellular mechanisms responsible for executing this process severely contributing to homeostasis in eukaryotic cells. Apart from cellular homeostasis, this system plays an essential role in maintaining neuronal functioning, regulation of chromatin structure, DNA repair, transcriptional regulation, cell cycle and cell division, synaptic development, maintaining synaptic connections and numerous other functions (Ciechanover, 2005). The UPS proteolytic pathway does this by the selective ATP-dependent degradation of target proteins such as short-lived, truncated or misfolded proteins by tagging these with polyubiquitin chains. In addition to the ubiquitination, other post transcriptional modifications such as SUMOylation and NEDDylation contribute to protein degradation (Dennissen et al., 2012) and autophagic processes are activated when the UPS capacity is exceeded (Lee et al., 2013; Nixon, 2013; Nilsson and Saido, 2014).

**Figure 3:**
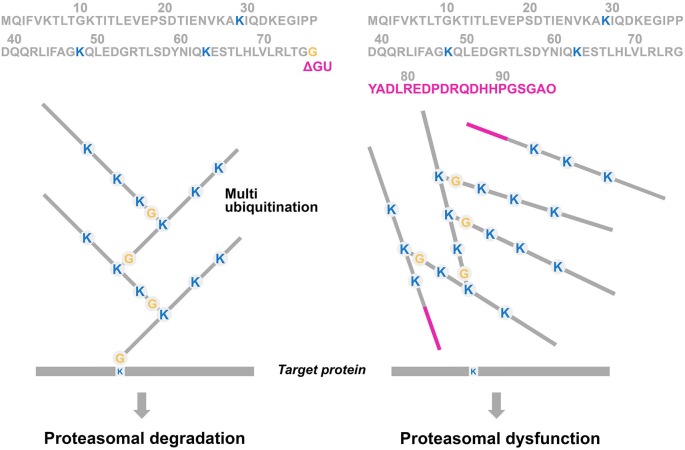
**Simplified scheme of the multiubiquitination process of proteasome substrates.** In the ubiquitin proteasome system (UPS), a substrate (= a degron, such as a misfolded protein) is (poly)ubiquitinated via a number of enzymatic steps (E1, E2 and E3). The C-terminal glycine (G76) of the UBB molecule will attach to lysine moieties (K) at positions 29, 48 and 63 which are involved in multiubiquitination and degradation. The UBB molecule “on top” of the target protein prone for degradation can be ubiquitinated itself, developing a multiubiquitination chain with at least four residues to be efficient for triggering proteasomal degradation (left panel). A GAGAG motif is present at the C-terminus of UBB and a dinucleotide deletion (ΔGU) occurs adjacent to this motif which results in an 20 amino acids extension (red bar), called UBB^+1^ (see Figure 4). This causes the absence of G76, necessary for binding to the target protein, and consequently it is not able to ubiquitinate (Ciechanover and Kwon, 2015). Interestingly, E3 enzymes are able to form a “forked” polyubiquitin chain in which two ubiquitin chains are linked ot adjacent lysines on a preceding ubiquitin moiety (e.g., K29, K48 and K63). These forked polyubiquitin chains are relatively resistant to degradation by the 26S proteasome (Kim et al., 2009).

Ubiquitin (UBB) is a highly conserved signaling molecule of 76 amino acids with a molecular mass of 8.5 kDa, that acts in the membrane, nucleus, and cytoplasm of all eukaryotic cells (Gregori et al., 1995). The main function of this protein is a post translational modification by covalent attachment via an isopeptide bond between UBB and a target protein which is called ubiquitination. The addition of a chain of UBB moieties on the target molecule is called multiubiquitination. The C-terminal glycine (G76) of the UBB molecule will attach to an internal lysine (K) of the target protein (Figure 3). After tagging the target protein with a polyubiquitin chain, the protein is subsequently translocated and degraded by a 26S proteasome complex in an ATP-dependent manner.

**Figure 4:**
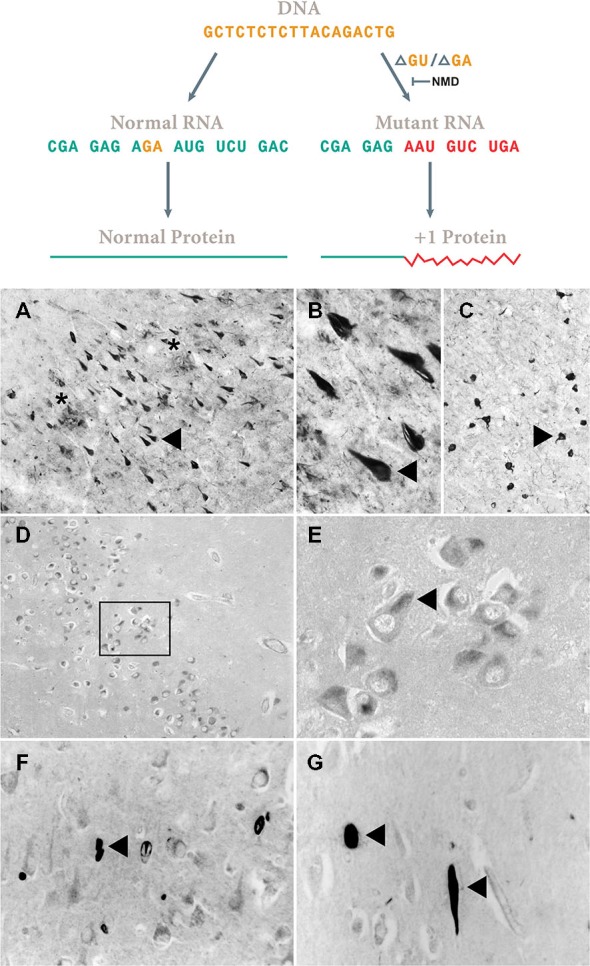
**Assembly-line slippage.** Loss of two bases [ΔGU, ΔGA and other ones (van Den Hurk et al., 2001)] in the mRNA garbles the rest of the sequence (Vogel, 1998). Non-sense mRNA decay (NMD) requires a downstream intron (Maquat, 2015) that is present in APP, Neurofilament H and MAP2b genes. However, in the UBB gene, this downstream intron is lacking and the misframed transcript apparently escapes NMD. As shown in Figure 3 thereby the essential C-terminus is lost. In **(A–C)** the resulting UBB^+1^ accumulation in neurofibrillary tangles (NFTs) (triangle) of the hippocampus of an AD patient and a similar one in Pick’s disease **(D,E)** in Pick bodies of CA1 in the hippocampus is shown. Note in **(A)** the presence of neuritic plaques (NPs) indicated by a star. **(F,G)** Neurofilament H transcripts apparently undergo a similar process (exon 1. aa. VGAARDSRAA), the resulting NFH^+1^ protein accumulates in CA1 of the hippocampus of AD patients (triangle). A similar reaction was found for MAP2B^+1^ (for details, see van Leeuwen et al., 2000). Of course these immunohistochemical data require many controls to avoid cross reactivity (Swaab et al., 1977) as was done for UBB^+1^. **(A–C)** 50 μm thick Vibratome sections, **(D–F)** 6 μm think paraffin sections.

**Figure 5:**
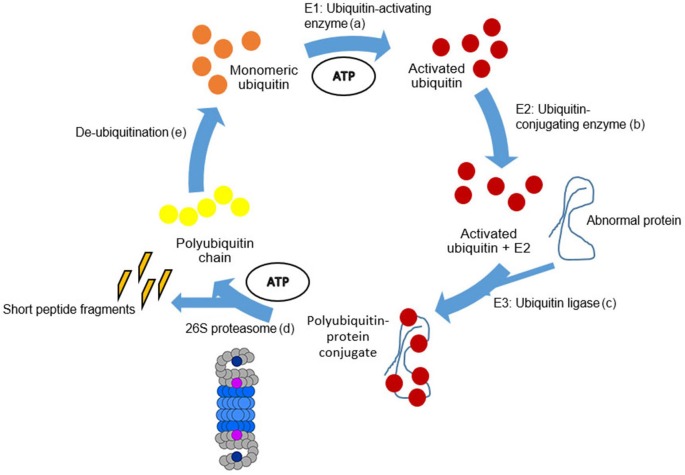
**Schematic representation of the UPS.** The degradation process by the UPS can be divided into five steps. (a) Beginning with monomeric ubiquitin (orange circles). Ubiquitin becomes activated in an ATP-dependent manner by E1. (b) Activated ubiquitin (red circles) is conjugated by E2 enzymes. (c) Thereafter, UBB is transferred to an internal lysine of the target protein by E3 ligases. Following, activated and conjugated ubiquitin binds to the abnormal protein forming the polyubiquitin-protein conjugate. (d) Subsequently, the polyubiquitin-protein conjugate is degraded by the 26S proteasome complex (Figure 5) by an ATP-dependent process (Peth et al., 2013). The abnormal protein is cleaved into short peptide fragments (orange pointed bars) and the polyubiquitin chain is released (yellow circles). (e) The polyubiquitin chain is split by de-ubiquitination enzymes into monomeric ubiquitins. For details, see Layfield et al. (2005).

The conjugation of UBB to the internal lysine residues (K) of the target protein is mediated by a cascade of E1-activating, E2-conjugating, E3-ligating and E4-elongating enzymes, which are stepwise described in Figure 5. UBB is generated from a precursor protein which is cleaved by ubiquitin C-terminal hydrolases (UCHs). UBB becomes activated in an ATP-dependent manner by the enzyme E1 via a high-energy thiolester bond between the carboxyl group of UBB and the active-site cysteine of E1 enzyme (Pickart, 2001). Subsequently, activated UBB is conjugated to the active site of an E2 ubiquitin-conjugation enzyme by a transthioesterification reaction. Currently, at least 30 different E2 enzymes have been described in the human genome (Bhowmick et al., 2013). Thereafter, UBB is transferred to an internal lysine of the target protein by E3 ubiquitin-protein ligases. There are at least 600 E3 ligases encoded in the human genome (Bhowmick et al., 2013). E3 ligases mediate the ligation between UBB and the target protein resulting in the ubiquitination of the target protein (Ciechanover and Kwon, 2015). Two different types of E3 enzymes are known: one type, the Homologous to E6-associated protein C-terminus (HECT) binds the E2-enzymes as well as the target protein and serves in this way as an intermediate docking station for UBB. A second type of E3-enzyme is the Real Interesting New Gene (RING) finger containing E3-ligase. In this instance, UBB is transferred directly from the E2-complex to the target protein by the RING-E3 ligase.

The multi-UBB chain linked with the lysine at position 48 (K48) has to be at least four residues long for efficient proteosomal targeting (Thrower et al., 2000). UBB has seven lysine residues indicating that diversity in polyubiquitin chain topology exists *in vivo* (Peng et al., 2003). As mentioned, the K48 bond involved in proteasome degradation is the most widely known and used topology. However, also different linkages exist like the K63 linkage which mediates non-proteolytic processes (Hadian et al., 2011) or the K11 linkage used for cell-cycle regulation and cell division (Matsumoto et al., 2010). The target protein undergoes several rounds of ubiquitination and in this way a polyubiquitin chain is formed (Ciechanover, 2014). Another, specialized type of E3 enzyme (E4, e.g., CHIP) is necessary for some substrates to be able to become polyubiquitinated to the desired length (Koegl et al., 1999; Hoppe, 2005).

UBB^+1^ is characterized as the first and only naturally occurring ubiquitin fusion degradation (UFD) substrate, is associated with neurodegeneration and ubiquitinated at K29 and K48 (Lindsten et al., 2002). UBB^+1^ is also ubiquitinated at K63 which opens the possibility of additional effects of UBB^+1^ (e.g., kinase activation; van Tijn et al., 2012). In summary, variation in the length of the polyubiquitin chain and the ubiquitin linkage location determine the fate of the ubiquitinated proteins, a process that is most probably more complex than previously assumed (Ciechanover, 2014; Ciechanover and Kwon, 2015).

**Figure 6:**
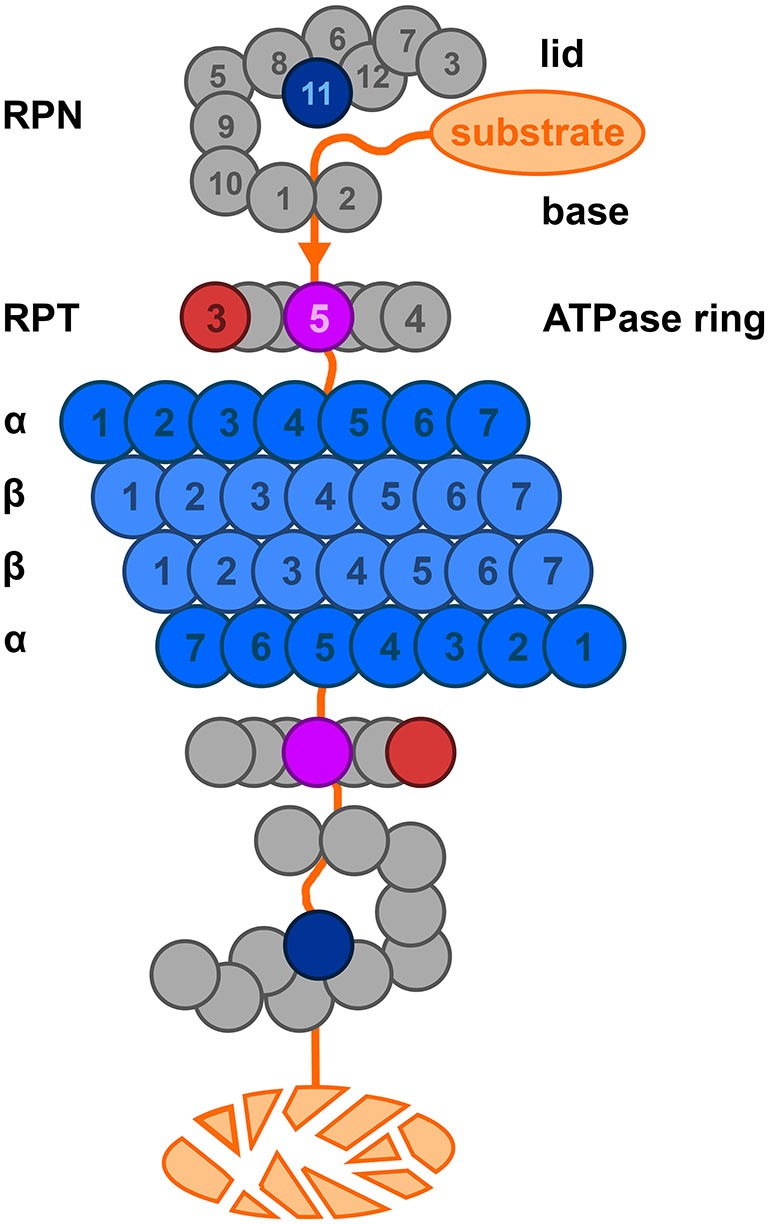
**A representation of the 26S proteasome complex based on Kostova and Wolf (2003).** The lid consists of eight non-ATP-ase subunits, the base consists of 6 ATP-ase and 3 non-ATP-ase subunits. Please note here that the yeast nomenclature is used. The catalytic core (blue) is a barrel-like structure and consists of four stacked heptameric rings. The function of many of the lid, base and ATPase ring subunits such as Rpt3 (red); Rpt 5 (purple); Rpn 11 (blue) have been described. For details, see text, Tsakiri and Trougakos (2015).

Subsequently, the polyubiquitin-protein conjugate is degraded by the 26S proteasome complex by an ATP-dependent process (Peth et al., 2013; Figure 6). The abnormal protein is cleaved into short peptide fragments of 6–10 amino acids (Ihara et al., 2012) and the polyubiquitin chain is released. The polyubiquitin chain is degraded by UCHL3 (Dennissen et al., 2011) and further processed by de-ubiquitinating enzymes (DUBs) into monomeric ubiquitin.

## The Proteasome

Polyubiquitinated proteins are translocated to the 26S proteasome which is described in detail in Figure 6. The proteasome has to fulfill a whole range of specialized functions: (1) cleavage of ubiquitin chains linked on the target protein; coined deubiquitination; (2) recognition and binding selectively to substrates prone to degradation; (3) chaperoning and unfolding of these substrates; (4) opening of the gates on each side of the 20S subunit; and (5) driving the substrate into the proteolytic core center in the 20S cylinder (Wolf and Hilt, 2004). We have performed an extensive immunohistochemical screen of almost all subunits of the proteasome in the hippocampal complex of AD and non-demented controls to see if a differential expression pattern of these subunits could be related to UBB^+1^ (Zouambia et al., 2008). Therefore the proteasomal complex is briefly introduced.

The proteasome is a cylindrical complex of 2.5 MDa which consists of two 19S caps and a proteolytic 20S core (Bedford et al., 2010; Ciechanover and Kwon, 2015). The 20S core complex has a barrel shape comprised of four rings and each ring includes seven subunits. The outer two rings are made up by 7 α-subunits facilitating docking of the 19S caps. The proteolytic chamber is composed of two inner rings consisting of β-subunits where β1, β2 and β5 show proteolytic active sites. The proteolytic activity can be specified in peptidyl-glutamyl-like (β1), trypsin-like (β2) and chymotrypsin-like (β5) activity. Alternatively, β1i, β2i and β5i can substitute for β1, β2 and β5 causing a different composition of the 20S complex which result in the immunoproteasome. These substitutions can be induced by pro-inflammatory (e.g., interferon-γ) cytokines resulting in immunoproteasomes, responsible for the generation of antigenic peptides presented by the MHC class I complex (Wolf and Hilt, 2004).

The 19S activator complexes consist of a base and a lid and are linked to each other via subunit regulatory particle non-ATPase 10 (Rpn10; S5a in mammals, please note that here the yeast nomenclature is used whereas aliases for the same subunit can be found at sites such as Online Mendelian Inheritance in Man (OMIM) or Genecards). The base consists of 6 ATPases of the AAA-family of proteins called Regulatory particle triple A proteins 1–6 (Rpt1–Rpt6), 3 additional non-ATPase subunits Rpn1 and Rpn2; and Rpn10/S5a (Zouambia et al., 2008; Ciechanover, 2014). Rpt 5 binds the ubiquitinated substrate. The lid on top of the base is built up out of eight different subunits (Rpn3, Rpn5 to Rpn9, Rpn 11 and Rpn12). Rpn11 contains a conserved metallo-isopeptidase motif which is necessary for deubiquitylation and proteolysis of substrates. It is responsible for the deubiquitination of the targeted substrate after this has been threaded into the 20S unit; for details, see Tsakiri and Trougakos (2015). It has been shown in immunohistochemical experiments that the Rpt3 subunit of the base is activated at the transcript level and colocalizes with UBB^+1^ in NFT of tauopathies including AD but not in synucleinopathies. This upregulation and colocalization of Rpt3 (or subunit 6b; human alias) with UBB^+1^ suggests a neuronal reaction to compensate for an increased need to degrade aberrant proteins (Zouambia et al., 2008).

Ultimately, as stated above, the proteasome degrades the substrate into short peptides and, thereby, reusable ubiquitin is released by DUBs. This deubiquitination process is tightly regulated by enzymes called deubiquitinating enzymes (at least 183 DUBS are known) which are partly essential for neuronal functioning. These DUBs can be subdivided into five different classes: (1) the UCHs; (2) the ubiquitin specific proteases (USPs); (3) the ovarian tumor proteases (OTUs); (4) the Machado-Joseph Disease protein domain proteases (MJDs); and (5) the JAB1/MPN/Mov34 metalloenzymes (JAMMs; Todi and Paulson, 2011). DUBs are responsible for cleaving ubiquitin precursors, rescuing target substrates from degradation, cleaving the ubiquitin chain at the proteasome entrance, controlling epigenetic mechanisms by deubiquitinating histones, and for disassembly of unanchored ubiquitin chains generating single ubiquitin moieties (Komander et al., 2009). Since ubiquitination is a reversible process, DUBs are responsible for maintaining the balance between free UBB and processed UBB (de Vrij et al., 2004; Dennissen et al., 2012). Recently it turned out that extended ubiquitin species like UBB^+1^ are potent DUB inhibitors leading to accumulation of proteins (Krutauz et al., 2014). This suggests that downregulation of UBB^+1^ is an attractive strategy to restore neuronal function.

## UPS in AD

There is compelling evidence that the UPS is impaired in many tauopathies such as AD and is involved in the accumulation of ubiquitinated proteins in AD brains (de Vrij et al., 2004; Ciechanover and Kwon, 2015). UBB^+1^ is formed during the transcription of the UBB gene by a mechanism that still has to be elucidated, but widespread DNA-RNA sequence differences do occur of which the mechanism is becoming clear (van Leeuwen et al., 1998; Gerez et al., 2005; Li et al., 2011, 2012; Wang et al., 2014). This process was coined molecular misreading by our group or RNA-DNA differences (RDD) by Cheungs group. UBB^+1^ accumulation is found in the neurons of all AD patients and it colocalizes with markers like MC1 and CP13 for NFTS, “kinky and curly” fibers, NTs and dystrophic neurites in plaques (van Leeuwen et al., 1998).

UBB^+1^ mRNA was present in all tested brain specimens of non-demented controls, tauo- and synucleinopathies, while the aberrant protein specifically accumulates not not only in all tauopathies investigated so far (e.g., AD; Fischer et al., 2003) but also in polyglutaminopathies (de Pril et al., 2004). Interestingly, the accumulation of the UBB^+1^ protein seems not to be initiated by an increase in molecular misreading, as the UBB mRNA concentration measured in cells from temporal cortices of AD patient aged between 54–88 is not increased, nor is it in non-demented controls aged between 38–90 (Gerez et al., 2005). So, question like the driving force of this process, the mechanism of molecular misreading and the difference in protein expression between tauopathies and synucleinopathies remain to be determined. A mechanism to check for errors in mRNA was described by the group of Maquat (Nagy and Maquat, 1998). Apparently UBB^+1^ transcripts escape mRNA surveillance as a downstream intron (>50 nucleotides downstream the mutation) is required which lacks in the UBB gene. This process is also called non-sense mediated RNA decay (NMD; Maquat, 2015).

So UBB^+1^ accumulates specifically in tauo- and polyglutaminopathies. Apparently, the UPS partly contributes to these phenomena:

a. Ubiquitination of UBB^+1^ is mediated by the enzymes E2-25K (Ko et al., 2010) and E3 ligases TRIP12 and HUWE1 (Park et al., 2009; Poulsen et al., 2012). E2-25K has an important role in providing a functional interaction between UBB^+1^ and Aβ toxicity (Ko et al., 2010).
b. UBB^+1^ chains seem to be resistant to disassembly by the DUB isopeptidase T, as UBB^+1^ lacks the C-terminal glycine residue (Lam et al., 2000).
c. Another explanation for UBB^+1^ accumulation is that it may be due to the inefficient hydrolysis of the C-terminus of UBB^+1^ by the DUB. UCHL3, possibly of the oxidative stress in neurodegenerative diseases (Dennissen et al., 2011).
d. Furthermore it has been shown that UBB^+1^ is a few amino acids too short to be efficiently degraded by the proteasome (Verhoef et al., 2009).
e. At low levels, the UBB^+1^ protein will be polyubiquitinated and degraded by the proteasome itself. However, at higher levels it will act as an inhibitor of the proteasome (right panel of Figure 3; van Tijn et al., 2007). Decreased proteasome activity has been shown in affected brain areas (e.g., hippocampus and cortex) of AD patients (Keller et al., 2000). The UPS exhibited a failure in proteasomal activity in cortical brain areas in AD (López Salon et al., 2000; Zouambia et al., 2008). Interestingly, the accumulation of Aβ in extracellular plaques may have an additional UPS inhibition, an effect shown in a cell free system (Gregori et al., 1995), in neuronal primary cultures (Lopez Salon et al., 2003) and in several AD tg mouse models (Almeida et al., 2006; Tseng et al., 2008). In addition, paired helical filaments (PHFs) isolated from AD brains are able to inhibit the proteasome via the 20S core (Keck et al., 2003). Moreover, the UPS inhibition not only increases the accumulation of UBB^+1^, but it also increases the deposition of hyperphosphorylated tau in NFTs (Morishima-Kawashima et al., 1993; Hol et al., 2005).
f. Earlier it was reported that UBB^+1^ causes neuritic beading, impairment of mitochondrial movements, mitochondrial stress and degeneration of primary neurons (Tan et al., 2007). The underlying mechanism may be due to mitochondrial dysfunction as we showed in a yeast strain that UBB^+1^ disturbs the UPS which causes mitochondrial stress and apoptosis. An unexpected enhancement of basic amino acid synthesis (arginine, ornithine and lysine) at mitochondria was induced by the expression of UBB^+1^ and this increase was identified as the decisive toxic event. This could be reversed by Cdc48/Vms1-mediated proteolysis which propose this pathway as a novel target for preventing neuronal dysfunction in AD (Braun et al., 2015).
g. It was shown that UBB^+1^ expression induces expression of heat-shock proteins (HSPs). This priming of the chaperone system promotes a subsequent resistance to oxidative stress (Hope et al., 2003).

These data suggest that the UPS is a central component in the pathogenesis of AD, a hypothesis recently firmly supported by other studies [Manavalan et al., 2013; International Genomics of Alzheimer’s Disease Consortium (IGAP), 2015].

To elucidate the effects of UBB^+1^ accumulation in the brain, we generated a tg mouse line (line 3413/donated to Jackson #008833) which shows overexpression of human UBB^+1^ in neurons of the postnatal brain, with strongest neuronal expression in the forebrain. In *in vivo* studies, a different behavioral phenotype from wild-type controls was found in the 3413 tg mice showing deficits in spatial memory retention in the Morris water maze (MWM) as well as deficits in context-dependent fear conditioning (FC) compared with WT mice. The proteome also showed changes compatible with AD (Fischer et al., 2009). Furthermore an unexpected result of line 3413 were changes in spontaneous breathing patterns and an altered response to hypoxic conditions as revealed in a functional analysis in the German Mouse Clinic. These data were anatomically supported by strong UBB^+1^ immunoreactivity in NFTs in brainstem regions of tg mouse line 3413 and validated in the same human AD brainstem regions such as the parabrachial nucleus (PBN) and the nucleus of the solitary tract (NTS; Irmler et al., 2012). It is suggested that brainstem dysfunction (e.g., respiration problems) is an early symptom as a result of UPS dysfunction. Indeed our data are consistent with respiratory symptoms also observed in AD patients (for details, see Irmler et al., 2012). This study confirms the power of a complete phenotypic screening of a tg line, such as was performed by the German Mouse Clinic. The focus in AD research has so far been on the entorhinal cortex, temporal cortex and hippocampus while the brainstem has been under investigated. Very recently, a study appeared showing significant total volume reduction and deformations in the brainstem of AD patients (Lee et al., 2015). These data together (e.g., cognitive and respiratory dysfunction) highlight the pivotal role of UBB^+1^ in basic mechanisms like homeostasis and breathing. According to Maslow’s hierarchy (Maslow, 1943), these basal levels needs to be fulfilled to jump to the next level of satisfaction.

## Suggestions for Experimental Follow Up Work

The topographic mapping of UBB^+1^ in the brain of mouse line 3413 with most intense site of UBB^+1^ accumulation in the forebrain and brainstem, show that line 3413 is not only useful for AD research (Gentier et al., 2015a) but also for studying other conformational diseases like HD and Familial encephalopathy with neuroserpin inclusion bodies (FENIB; Schipanski et al., 2014). This FENIB tg line was crossed with line 3413 and resulted in an effect on endoplasmic reticulum associated degradation (ERAD). Line 3413 was also useful when crossbred with mouse Line 85 [carrying the Swedish double mutation (K594M/N595L)] and human PS1 with a deletion of exon 9 (APPPS1; Jankowsky et al., 2004) regarding the modulation of the Aβ42 plaque load at younger age and the unexpected rise in γ-secretase activity (Gentier et al., 2015b).

The contribution of the UPS compounds with regard to degrading UBB^+1^ can be addressed in yeast around three objectives. (1) Which are the lethal variants of UBB^+1^? (2) What is the role of the AAA-ATPases Cdc48/VCP and Rpt1–6 in degrading UBB^+1^? and (3) What is the role of DUBs in modifying UBB^+1^ and modulating its cytotoxicity?

## Conflict of Interest Statement

The authors declare that the research was conducted in the absence of any commercial or financial relationships that could be construed as a potential conflict of interest.

## Abbreviations

Aβ: amyloid β
AICD: APP intracellular domain
ADAM10: A Disintegrin and metalloproteinase domain-containing protein 10
AD: Alzheimer’s disease
APP: amyloid precursor protein
APPs: soluble N-terminal part of APP
APP-CTF: C-terminal part of APP
DS: Down syndrome
DUB: deubiquitinating enzyme
EOAD: early-onset AD
ERAD: endoplasmic reticulum associated degradation
FAD: familial Alzheimer’s disease
FC: fear conditioning
FENIB: familial encephalopathy with neuroserpin inclusion bodies
G: glycine
GWAS: genome wide association studies
HD: Huntington’s disease
HECT: homologous to E6-associated protein C-terminus
HSP: heat-shock proteins
JAMM: JAB1/MPN/Mov34 metalloenzyme
LOAD: late-onset AD
K: lysine
MCI: mild cognitive impairment
MJD: Machado-Joseph Disease protein domain protease
MWM: Morris water maze
NFT: neurofibrillary tangle
NMD: non-sense mediated RNA decay
NP: neuritic plaque
NT: neuropil thread
NTS: nucleus of the solitary tract
OTU: ovarian tumor proteases
PBN: parabrachial nucleus
PD: Parkinson’s disease
PHF: paired helical filament
PS1: presenilin 1
PS2: presenilin 2
RDD: RNA-DNA differences
RING: really interesting new gene
RPT: regulatory particle triple
SAD: sporadic Alzheimer’s disease
tg: transgenic
TREM2: triggering receptor expressed on myeloid cells 2
UBB: ubiquitin
UBB^+1^: ubiquitin B^+1^
UCH: ubiquitin C-terminal hydrolase
UFD: ubiquitin fusion degradation
UPS: ubiquitin proteasome system
USP: ubiquitin specific proteases.
